# What is beyond testicular torsion and epididymitis? Rare differential diagnoses of acute scrotal pain in adults: A systematic review

**DOI:** 10.1016/j.amsu.2020.05.031

**Published:** 2020-05-29

**Authors:** Nadine Sieger, Francesca Di Quilio, Jens-Uwe Stolzenburg

**Affiliations:** a Department of Urology, University Hospital Leipzig, Germany; b Department of Urology and Kidney Transplantation, Martin Luther University, Halle (Saale), Germany

**Keywords:** Acute testicular pain, Acute scrotal pain, Epididymo-orchitis, Urological emergency, Segmental testicular infarction

## Abstract

**Background:**

Acute scrotal pain is a urological emergency. While for testicular torsion and acute epididymitis clinical recommendations are well established, few is known about low incidence causes of acute scrotal pain. Our aim is to identify and characterise rare differential diagnoses of acute scrotal pain in order to give diagnostic and therapeutic recommendations.

**Materials and methods:**

A systematic literature search was performed in PubMed, Web of Science and the Cochrane Library databases up to February 2019 according to the Preferred Reporting Items for Systematic Reviews and Meta-Analysis (PRISMA) statement. The systematic review protocol was registered on PROSPERO (CRD42018099472).

**Results:**

Eighty-four publications were selected for analysis. The databases provided mostly case reports, series and small studies, overall reporting on a cohort of 245 cases. Tumors, segmental testicular infarction, testicular vasculitis, pancreatitis, brucellosis, spermatic vein thrombosis, acute aortic syndrome and appendicitis were identified as rare underlying causes of acute scrotal pain and were characterised. As a result of our data analysis we were able to draw an overview of the rare differential diagnoses and diagnostic management of acute scrotal pain.

**Conclusion:**

Rare differential diagnoses of acute scrotal pain are susceptible to misinterpretation as testicular torsion or acute epididymo-orchitis. Surgical management is indicated in case of suspicion for torsion or tumor. We herein present knowledge of the rare differential diagnoses and raise awareness for associated systemic disease in order to facilitate disease management and increase the potential for testicle-sparing treatment.

## Highlights

• First systematic approach to assess literature for rare differential diagnoses of acute scrotal pain.
• Disease characterization including sonographic features.
• Flow chart for the diagnostic and treatment options of acute scrotal pain beyond testicular torsion and common epididymo-orchitis.

## 1 Introduction

Acute scrotal pain is one of the leading symptoms causative for presentation at the urological emergency department. Testicular torsion and acute epididymitis are a major subject and can be identified in the majority of cases by medical history taking, clinical examination and scrotal ultrasound. Suspicion of testicular torsion is an indication for urgent surgical exploration. Patients with acute epididymitis should be subjected to antibiotic therapy according to the most probable pathogen and local anti-infective resistance pattern [1]. Although not typically associated with acute scrotal pain, testicular tumors have been reported to be accompanied by scrotal pain in up to 27% of cases. Ultrasound sensitivity in the detection of testicular tumor is approximately 100% and once diagnosed, the diagnostic and therapeutic management is clearly defined by guidelines [2,3]. Yet, presence of sonographic testicular lesions due to pathologies other than tumors are a major challenge in the evaluation of acute scrotal pain.

The aim of this review is to provide an overview and detailed description of rare differential diagnoses of acute scrotal pain in order to give clinical recommendations. We seek to identify scrotal pathologies that are at risk to be confused with testicular torsion and common acute epididymitis.

## 2 Materials and Methods

**Fig. 1:**
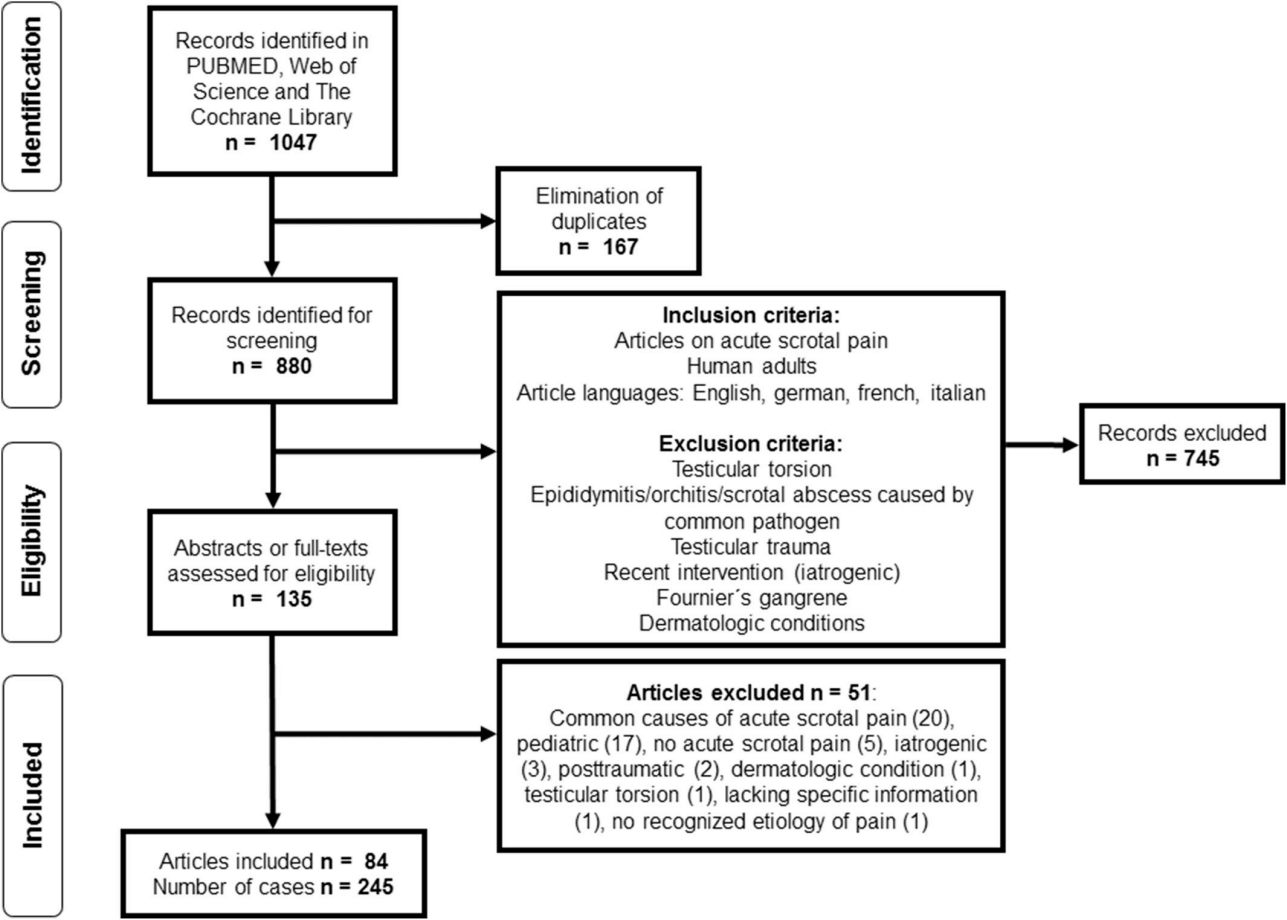
PRISMA Flow chart - Selection of publications.

A systematic literature search for acute scrotal pain was performed in March 2017 on PubMed, Web of Science and The Cochrane Library databases. An update of the search was performed on February 11, 2019. We applied the following search strategies: scrot*[Title/Abstract] OR testic*[Titel/Abstract] AND pain AND acute, (scrotal OR testicular AND pain) AND acute (Title) and acute scrotal pain, respectively. The systematic search has been in line with the Preferred Reporting Items for Systematic Reviews and Meta-Analysis (PRISMA) Guidelines [4] and was registered on PROSPERO International prospective register of systematic reviews (CRD42018099472) before data extraction. We particularly searched for differential diagnoses masquerading as testicular torsion or acute epididymitis/epididymo-orchitis. Articles on acute scrotal pain as a result of trauma, interventions, dermatologic pathologies and Fournier's gangrene were excluded as they were less likely to simulate torsion or epididymitis. The search strategy including a detailed overview of the inclusion/exclusion criteria is indicated in Fig. 1. After eliminating duplicates and screening of titles, two independent authors assessed abstracts and full texts in order to extract relevant literature. Disagreements between the two authors were resolved by discussion and consensus.

Selected articles were grouped for the different disease entities. A standardised form was used to extract the following data from the articles: study design, patient age, pertinent medical history, symptoms, clinical findings, ultrasonography findings, treatment and interventions, disease course/outcome.

## 3 Results and discussion

### 3.1 Literature search results and data extraction

**Fig. 2:**
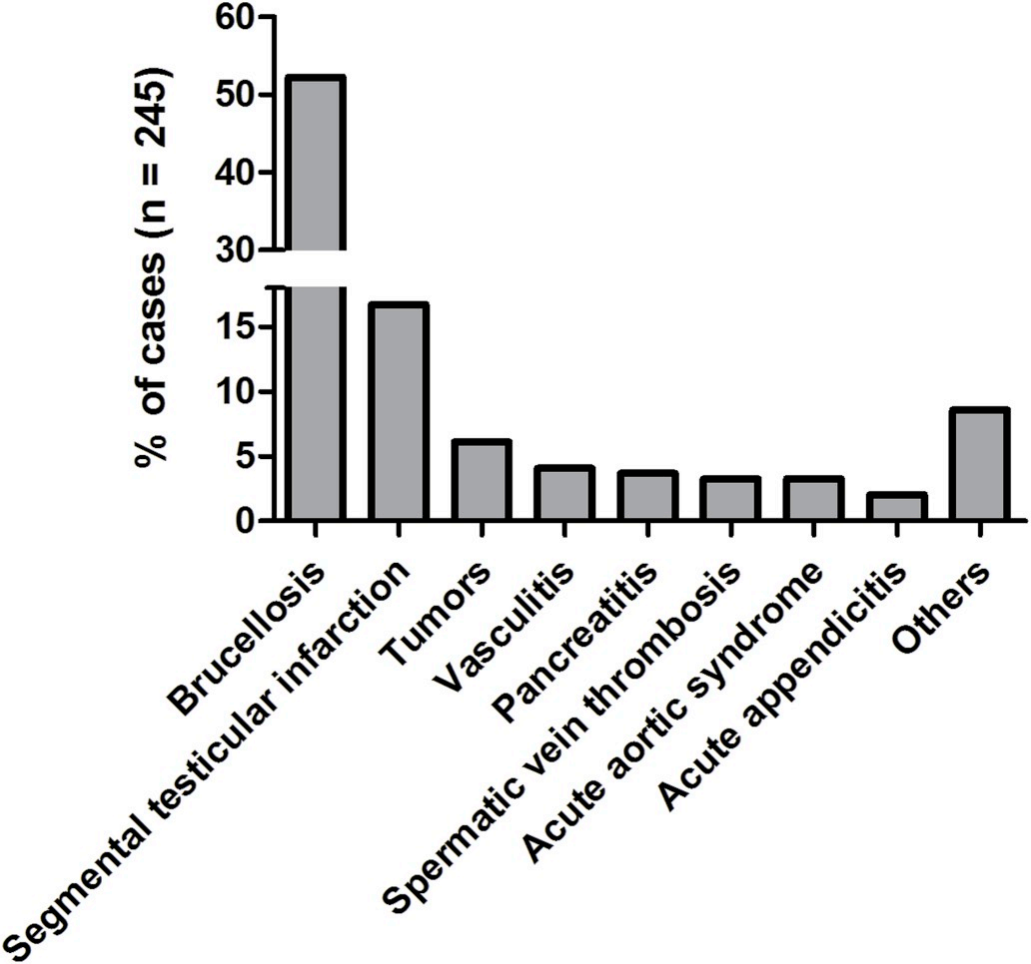
Representation of rare causes of acute scrotal pain in the literature. Total number of cases n = 245.

**Fig. 3:**
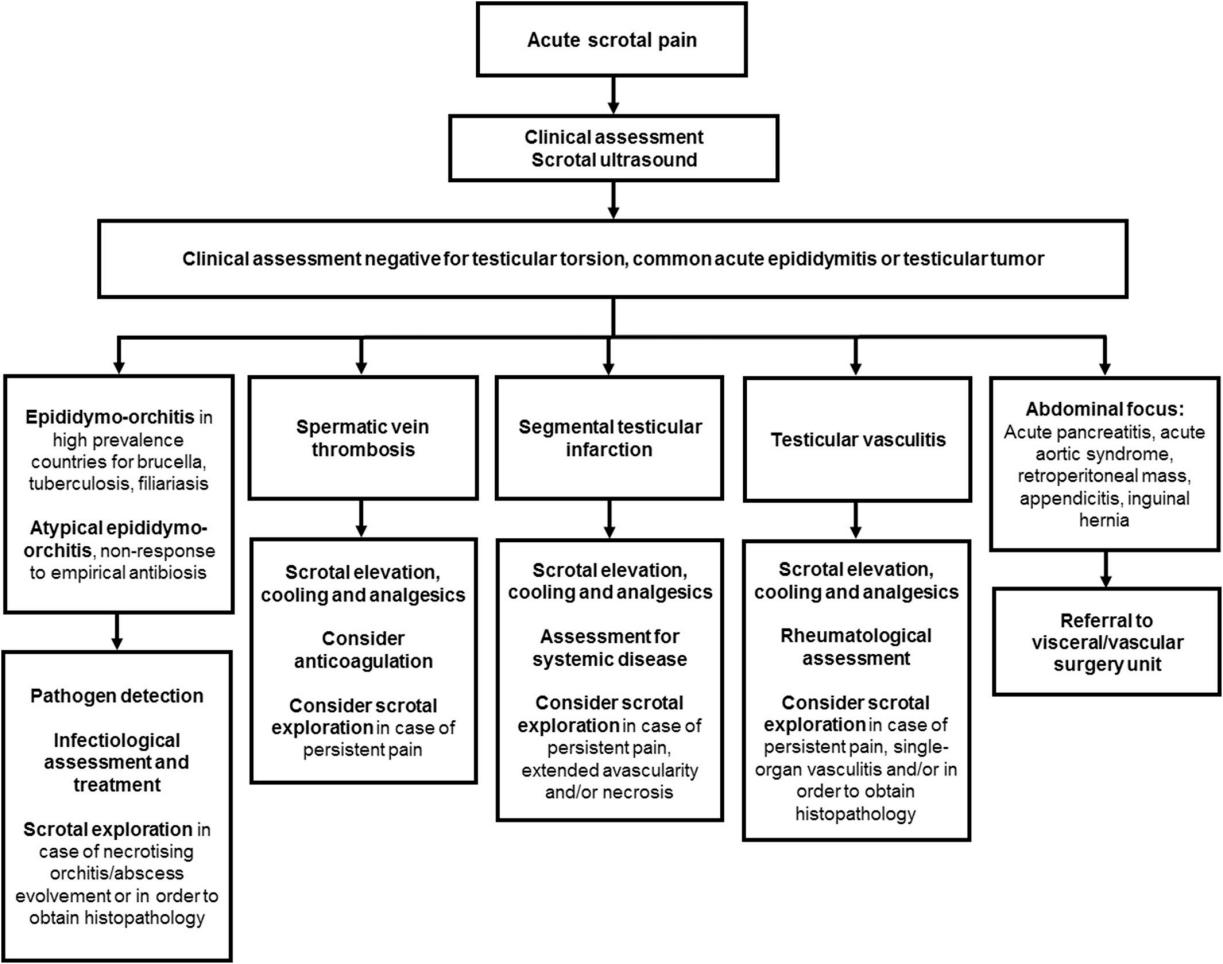
Differential diagnoses after exclusion of the most common causes of acute scrotal pain and diagnostic and treatment options as suggested by the literature.

Eighty-four publications were included according to our search criteria (Fig. 1). Tumors, segmental testicular infarction, testicular vasculitis, acute pancreatitis, brucellosis, plexus pampiniformis/spermatic vein thrombosis, acute aortic syndrome, appendicitis, tuberculosis and filariasis were identified as rare underlying pathologies of acute scrotal pain. Quantitative representation of cases is shown in Fig. 2. We identified five retrospective studies, one prospective study, one review article and 77 case reports and series, overall reporting on 245 cases. Articles and case characteristics are described in Table 1, Table 2. A selection of sonographic characteristics is depicted in Table 3. An overview of rare differential diagnoses and clinical management as suggested by the literature is provided in Fig. 3.

**Table 1:** Selected publications and clinical findings.

Number of articles [References]	Number of cases (age)	Side of acute scrotal pain	Additional symptoms and findings	Associated factors and comorbidity	Treatment and interventions (Classification)
Tumors					
12 [[5], [6], [7], [8], [9], [10], [11], [12], [13], [14], [15], [16]]	15 (mean 38 ± 12.3)	Right n = 8 Left n = 5 Not mentioned n = 2	Intrascrotal mass n = 9 Concomitant hydrocele n = 3 Others (fever, fatigue, weight loss, abdominal pain etc.)	Not mentioned n = 7 None n = 4 History of cryptorchidism n = 2 [6,10] Parks-Weber syndrome n = 1 [7] Asbestos exposure n = 1 [13]	**Orchiectomy** n = 6 (embryonal carcinoma [5], choriocarcinoma with hematoma [8], seminoma with purulent orchitis [7], epidermoid cyst [16]) **Orchiectomy**, **chemotherapy** n = 1 (Non-Hodgkin-Lymphoma) [11] **Orchiectomy, chemotherapy**, **radiotherapy** n = 1 (granulocytic sarcoma) [10] **Orchiectomy**, **radiotherapy** n = 1 (seminoma) [6] **Orchiectomy**, **resection of tunica vaginalis** n = 1 (mesothelioma of the tunica vaginalis) [13] **Resection of tunica vaginalis** n = 2 (mesothelioma and hydatid torsion [14], renal cell carcinoma metastasis [12]) **Resection of epididymis** n = 2 (adenomatoid tumor of the epididymis) [15] **Chemotherapy** n = 1 (choriocarcinoma with metastasis) [9]
**Segmental testicular infarction**					
11 [[17], [18], [19], [20], [21], [22], [23], [24], [25], [26],^69^]	41 (18–90)	Left n = 21 Right n = 11 Not mentioned n = 7 Bilateral n = 2	No palpable lesion n = 21 None n = 7 Scrotal swelling n = 6 Not mentioned n = 6 Flank pain n = 1	None n = 23 Sickle cell disease n = 3 [17,23,69] Cardiovascular disease n = 3 [21,24,69] Others (nicotine abuse [24], hand foot mouth disease [18] etc.)	**Conservative** n = 25 [21,26,69] **Partial orchiectomy** n = 8 [[17], [18], [19], [20]] **Orchiectomy** n = 7 [18,[21], [22], [23]] **Bilateral scrotal exploration** n = 1 [25]
**Testicular vasculitis**					
9 [27,[29], [30], [31], [32],[70], [71], [72], [73]]	10 (mean 39.9 ± 17.7)	Right n = 5 Left n = 1 Bilateral n = 2 Not mentioned n = 2	Scrotal swelling n = 5 Purpura/skin nodules/skin necrosis n = 4 Fever n = 3 Weight loss n = 3 Arthralgia n = 3 None n = 2 Paresthesia n = 1 Myalgia n = 1 Ophthalmoplegia n = 1 Loss of vision n = 1	None n = 3 Thrombosis n = 2 [29,72] Raynaud syndrome n = 2 [32,73] Others (acute myeloid leukemia [72], end-stage renal disease [29], prior Hepatitis B vaccination [71] etc.)	**Orchiectomy**, **immunosuppressants** n = 3 (Polyarteriitis nodosa) [30,72] O**rchiectomy** n = 2 (testicular single-organ vasculitis) [31,32] **Bilateral orchiectomy, skin biopsy**, **immunosuppressants** n = 1 (PAN) [29] **Bilateral scrotal exploration**, **testis biopsy**, **glucocorticoids** n = 1 (PAN) [73] **Skin biopsy**, **glucocorticoids** n = 1 (Schönlein-Henoch purpura) [70] **Renal biopsy**, **immunosuppressants** n = 1 (PAN) [71] **Glucocorticoid treatment only** n = 1 (PAN) [72]
**Acute pancreatitis**					
9 [[33], [34], [35], [36], [37],^39^,^40^,^74^,^75^]	9 (mean 37.4 ± 8.7)	Left n = 7 Right n = 2	Scrotal swelling n = 9 Abdominal pain n = 7 Scrotal erythema/discoloration n = 3 Nausea/vomiting n = 2	Alcohol abuse n = 7 Others (nicotine abuse [36], ulcera ventriculi [39])	**Percutaneous drainage, antibiotics**, **fasting** n = 2 [33,34] **Scrotal exploration** n = 2 [35,40] **Conservative** n = 2 [74,75] **Scrotal exploration/drainage, laparotomy, necrosectomy** n = 3 [[36], [37], [39]] (Exitus letalis n=1) [39]
**Brucellosis**					
7 [38,[41], [42], [43], [44], [45],^76^]	128	Unilateral 96% Bilateral 3% Not mentioned 1%	Scrotal swelling 98% Fever 79% Arthralgia 41% Urinary tract symptoms 27% Hepato(spleno-)megaly 20% Headache 9% Weight loss 7% Vomiting 5%	Country/region endemic for Brucellosis 100% Consumption of raw milk products 70% Occupational exposure 35%	**Combined antibiotic therapy** 100% **Orchiectomy** 5% [41,43,45]
**Spermatic vein thrombosis**					
7 [[46], [47], [48], [49],[77], [78], [79]]	8 (mean 39.4 ± 12.5)	Left n = 5 Bilateral n = 2 Right n = 1	Scrotal/scrotoinguinal swelling/mass n = 7 Varicocele n = 3 Urethritis n = 1 Inguinal hernia n = 1	History of vasectomy n = 2 [48,49] History of orchidopexy [77] Others (drug abuse [77], Protein C deficiency [79] etc.)	**Anti-inflammatory drugs, antibiotics**, **anticoagulation** n = 2 [77,79] **Anti-inflammatory drugs** n = 1 [78] **Excision of thrombosed vein/varicocele** n = 3 [[46], [47], [48]] **Excision of thrombosed vein**, **orchiectomy** (left side) **antibiotics**, **anticoagulation** (right side) n = 1 [48] **Varicocelectomy**, **inguinal hernia repair** n = 1 [49]
**Acute aortic syndrome**					
5 [[50], [51], [52], [53],^80^]	8 (mean 68.6 ± 13.1)	Left n = 6 Right n = 2	Abdominal pain/pulsating mass n = 3 Vomiting n = 2 Fever n = 2 Hypertension n = 2 None n = 2 Hypotension n = 1	Hypertension n = 5 Cardiovascular disease n = 2 [50] Nicotine abuse n = 2 [51,53] Diabetes mellitus n = 1 [50]	**Laparotomy/aortic graft placement** n = 6 [[50], [51], [52], [53],^80^] (Exitus letalis n = 2) [[50], [52]]
**Acute appendicitis**					
5 [[54], [55], [56], [57], [58]]	5 (9–61)	Right n = 4 Left n = 1	Scrotal swelling n = 1 Scrotal erythema n = 2 Abdominal pain n = 2 Vomiting n = 2 Fever n = 1 None n = 1	Nicotine abuse n = 2 [56,58] Others (history of right inguinal hernia repair [57] etc.)	**Appendectomy** n = 3 [[54], [55], [56]] **Appendectomy with right inguinal hernia repair** (appendicitis within an inguinal hernia n = 2 [57,58])

**Table 2:** Other rare causes of acute scrotal pain. Selected publications and clinical findings.

Author et al.	Number of cases (age)	Classification	Side of acute scrotal pain	Additional symptoms and findings	Associated factors	Treatment and interventions
**Tuberculosis**						
Guler (2006) [61]	1 (54)	Tuberculous epididymitis	Right	Epididymal swelling, whitish discharge, fever, sudor, weight loss, Addison's disease	Recurrent epididymitis	Bilateral epididymectomy, antituberculous treatment
Khan (2015) [63]	1 (35)	Tuberculosis of tunica albuginea and vaginalis	Left	Scrotal swelling and erythema, hydrocele	Country endemic for tuberculosis (Pakistan)	Surgical exploration, hydrocelectomy, antituberculous treatment
Kinnear (2016) [62]	1 (18)	Tuberculous epididymo-orchitis and abscess	Left	Malaise	Endemic country (Afghanistan)	Abscess incision, drainage, antituberculous tratment
**Filariasis**						
Di Tonno (2010) [64]	1 (25)	Filariasis of the epididymis	Right	Epididymal enlargement and erythema	Tropical region (Bangladesh)	Scrotal exploration, removal of epididymal nodule, referral to infectious diseases unit
Mussner (1997) [65]	1 (38)	Filariasis of the funiculus spermaticus	Left	None	Endemic region (Nepal)	Scrotal exploration, abscess drainage, funiculus biopsy, filaricide medication
Vashisht (2018) [81]	1 (37)	Testicular filariasis	Bilateral	Fever, joint pain	Endemic region (India)	Filaricide medication
**Emphysematous epididymo-orchitis**						
Mandava (2014) [66]	1 (51)	Emphysematous epididymo-orchitis	Right	Scrotal swelling and erythema	Diabetes mellitus	Orchiectomy, surgical depridement, antibiotics
Yen (2016) [67]	1 (69)	Emphysematous epididymo-orchitis	Right	Fever	Rectum cancer with prostate invasion	Orchiectomy, debridement, antibiotics
**Inguinal hernia**						
Desai (2012) [59]	1 (48)	Inguinal hernia resulting in testicle ischemia	Right	Large scrotoinguinal mass and edema	One-year history of right-sided inguinal hernia	Hernia reposition and repair
Mouli (2010) [60]	1 (65)	Acute vesicoinuginal hernia	Right	Large scrotoinguinal mass, urinary retention	Not mentioned	Surgical exploration, bladder reposition, inguinal hernia repair
**Ureteral stone**						
McGee (1993) [52]	1 (44)	Ureteral stone	Left	Lower abdominal pain, nausea, vomiting	Not mentioned	Not mentioned
Wachsberg (2017) [68]	3 (24–46)	Distal/midureteral stone	Right n = 2 Left n = 1	Not mentioned	Not mentioned	Not mentioned
**Others**						
Altiparmak (2003) [82]	1 (60)	Epididymal cysts associated with adult polycystic kidney disease (APKD)	Left	Testicular swelling, bloody ejaculate, seminal vesicle cysts	APKD, psoriasis vulgaris	Not mentioned
Birkan (2016) [83]	1 (16)	Torsion of the epididymis	Left	Scrotal swelling and edema	None	Patient refused surgery
Chang (2015) [84]	1 (34)	Idiopathic lymphocytic orchitis	Left	Not mentioned	Common cold	Antibiotics, corticosteroids, orchidectomy
Hikosaka (2008) [85]	1 (25)	Torsion of spermatocele	Left	Palpable mass above the testicle	None	Scrotal exploration, spermatocele resection
Karanikas (2018) [86]	1 (31)	Greater omental torsion	Right	Abdominal pain, vomiting, fever	Inguinal hernia repair during childhood	Laparotomy, dissection of twisted omentum
Nana (2014) [87]	1 (70)	Left renal vein thrombosis	Left	Scrotal swelling, left loin pain	Chronic liver disease	Anticoagulation
Sountoulides (2007) [88]	1 (22)	Arteriovenous malformation of the spermatic cord	Right	None	Recurrent scrotal pain	Scrotal exploration, orchiectomy
Takeuchi (2017) [89]	1 (21)	Idiopathic intratesticular hemorrhage	Left	Testicular swelling, left lower abdominal pain	Not mentioned	Scrotal exploration, orchiectomy

**Table 3:** Sonographic characteristics for rare differential diagnoses of acute scrotal pain.

Pathology (Number of cases depicted by scrotal ultrasound)	General characteristics	Echogenicity	Perfusion in Color Doppler
**Brucellosis** (n = 54)	Testicular enlargement [38,[41], [42], [43],^45^] Epididymal enlargement [42,45] Testicular abscess [43] Hydrocele [38,[41], [42], [43]]	Hypoechogenic testis [43,45] Hypoechogenic testicular lesions [38,41,43,45] General changes in echotexture [41,42]	Testicular hypervascularity [43]
**Segmental testicular infarction** (n = 41)	Oval/rounded [21,26,69] or wedged-shaped [19,21,69] testicular lesions Well [19,20] or poorly defined [23] margins	Hypoechogenic lesions [17,20,22,24,25] Isoechoic lesion [21] Mixed echogenic lesions [19,23]	Avascular lesions [17,[20], [21], [22],[24], [25], [26],^32^,^69^] Hypovascular lesions [21,23,25]
**Testicular vasculitis** (n = 9)	Testicular lesions without mass effect [71] Oval shaped lesion [31] Enlarged testis [72] Henoch-Schönlein purpura: Scrotal skin thickening, epididymal enlargement, hydrocele [70]	Mixed echogenic lesions [31,72] Hypoechogenic lesion [71]	Avascular lesions [[29], [30], [31],^71^,^73^] Hypovascular lesions [29,72]
**Acute pancreatitis** (n = 6)	Scrotal wall edema [33,36,37] Edematous testicle [35] Fluid collection around spermatic cord and epididymis [75] Extratesticular mass (phlegmon) [37] Hydrocele [40]	Normal testicular echogenicity [33,36,37]	Intact testicular blood flow [37,40,75]
**Spermatic vein/varicocele thrombosis** (n = 5)	Endoluminal thrombus in the pampiniform plexus/spermatic vein [46,49,[77], [78], [79]] Thrombosed varicocele [49,79] Hydrocele [77]	Hypoechoic thrombus [46,49,77]	Endoluminal filling defect with absent blood flow [[46], [49], [77], [78], [79]]
**Tuberculous epididymitis** (n = 2)	Enlarged epididymal tail [61,62] Scrotal abscess: Well-defined fluid collection [62]	Heterogeneous and hypoechogenic epididymis [61,62]	Hypervascular epididymis [62]
**Emphysematous epididymo-orchitis** (n = 2)	Enlarged, ill-defined testis and epididymis [66]	Hypoechogenic testis and epididymis [66] with multiple reflective, hyperechogenic foci suggestive of gas shadows [66,67]

### 3.2 Testicular and non-testicular intrascrotal tumors

Testicular tumors usually present as painless masses. We identified testicular cancer [[5], [6], [7], [8], [9]], granulocytic sarcoma [10], intratesticular manifestation of Non-Hodgkin-Lymphoma [11], intrascrotal metastasis of renal cell carcinoma [12], mesothelioma [13,14], adenomatoid tumor of the epididymis [15] and intratesticular epidermoid cyst [16] as potential sources of acute scrotal pain (Table 1).

Tumor infarction, necrosis and space-occupying growth are recognised sources of pain. Tumors displaying these properties have the potential to cause acute scrotal pain. In our selection of cases pain ocurred due to arterial bleeding and hematoma [8], associated infection [6,7] and hydatid torsion [14] which favored early detection of the tumor. On the other hand, epididymo-orchitis masking an embryonal carcinoma resulted in delayed orchiectomy [5]. Therefore, it seems crucial to consider tumors as differential diagnoses of acute scrotal pain.

### 3.3 Segmental testicular infarction

Eleven articles reporting on segmental testicular infarction, including one retrospective study, two case series and eight case reports, were identified. Overall, 41 cases were reported. Characteristics are shown in Table 1. Areas of infarction were characterised by lack of flow in color Doppler images (Table 3). Association with cardiovascular disease, sickle cell disease, vasculitis, epididymo-orchitis, former intervention or trauma suggests a role of these factors but in the majority of patients the etiology was considered idiopathic (n = 23). 25 Patients received conservative analgesic treatment. Eight patients underwent partial orchiectomy as the result of scrotal exploration [[17], [18], [19], [20]]. Seven patients underwent orchiectomy [18,[21], [22], [23], [24]]. Histopathological examination revealed hemorrhagic infarction with occluding thrombosis of segmental testicular vessels and presence of necrotic tissue [[18], [19], [20],^23^,^24^]. Follow-Up was reported in three articles for patients, who had undergone testicle-preserving treatment, and showed significant reduction in lesion size compared with earlier examinations [21,25,26].

Conservative therapy is a valid option in segmental testicular infarction. In doubtful cases, surgical exploration is indicated. Frozen-section, if available, is an option to enable testicle-sparing treatment. Extensive infarction or persistent pain may be an indication for orchiectomy.

### 3.4 Testicular vasculitis

Testicular vasculitis usually occurs as part of a systemic vasculitis but can appear as single-organ vasculitis if restricted to the testis. Pannek et al. described an incidence of acute scrotal pain of 2–8% in patients with Polyarteritis nodosa (PAN), 7% in Schönlein-Henoch purpura and 4–31% in Behҫet's disease [27]. Testicular pain is incorporated in the American College of Rheumatology 1990 criteria for the classification of PAN [28]. Our literature review revealed ten cases of vasculitis associated with acute scrotal pain (Table 1). Seven of ten patients were initially suspected of testicular torsion or tumor and underwent surgical intervention, six of them were orchiectomised. Extended necrosis of the testes was seen upon exploration in two patients [29,30]. Histopathologic examination showed fibrinoid necrosis and thrombi of medium-sized arteries associated with inflammatory infiltrate within multiple areas of hemorrhagic infarction, as well as presence of giant cells [31,32]. Seven cases fulfilled the criteria for PAN. All but the two patients with single-organ vasculitis received systemic immunosuppressive treatment.

The value of orchiectomy in testicular vasculitis needs to be considered case by case. Focal hypo- and avascularity as seen in scrotal ultrasound are in keeping with testicular infarction, which is secondary to vascular destruction (Table 3). The indication for orchiectomy depends on the extend of testicular destruction and persistence of complaints. Single-organ vasculitis can be treated by surgical removal of the affected site, which by some authors has been considered even more effective than systemic treatment [31]. However, conservative management of testicular vasculitis is a valid option. All patients with suspicion of vasculitis shoud be referred to a rheumatologist in order to check indication for systemic therapy.

### 3.5 Acute pancreatitis

Presentation of pancreatitis with acute scrotal pain has been described in nine case reports (Table 1). Clinical diagnosis was determined by symptomatology, laboratory values (elevated serum amylase, lipase, leukocytosis), abdominal Computed tomography findings showing pancreas inflammation and retroperitoneal fluid accumulation extending through the inguinal channel down to the scrotum [[33], [34], [35], [36], [37], [38]] and/or surgical exploration. In five cases, onset of scrotal pain was delayed by one to seven days after onset of abdominal symptoms. Remarkably, two patients had isolated scrotal pain. Scrotal exploration was performed in four patients under the suspicion of testicular torsion revealing destructive and necrotic tissue inflammation and fluid secretion [35,36,39,40]. Laparotomy was performed in three cases of necrotising pancreatitis showing a retroperitoneal path of necrotic tissue descending from the pancreas down to the testis [36,37,39]. Two patients received percutaneous drainage of retroperitoneal fluid collections [33,34]. Recovery was achieved in all but one patient who died of septic toxic multiple organ failure [39].

Acute scrotal pain as a manifestation of acute pancreatitis is caused by retroperitoneal pancreatic fluid descensus. Awareness of this pathomechanism and complete diagnostic investigation including medical history/alcohol anamnesis, laboratory values and abdominal sonography/CT in a multi-disciplinary approach may aid to determine prompt diagnosis in this potentially life-threatening condition.

### 3.6 Brucellosis

Brucellosis is a systemic, bacterial zoonosis that has been described to involve testicles, epididymis, seminal vesicles and prostate in 2–20% [41,42]. In their prospective study Akinci et al. determined an incidence of 12.7% for epididymo-orchitis in patients with brucellosis [43]. Our literature search identified one prospective study, four retrospective studies and two case reports on Brucella epididymo-orchitis. In total, 128 cases were reported. Characteristics are depicted in Table 1. Scrotal pain occurred at different stages of systemic Brucellosis manifestation: As first manifestation of the disease (32%) [41,42,44], simultaneously at disease onset (24%) [38,41,42], after systemic disease onset during treatment (10%) [41,43], following prior treatment of Brucellosis (2%) [43,45], as far as reported. Diagnosis of Brucellosis was determined by clinical evaluation, including symptomatology, blood cultures (positive in 48% of cases), Brucella serology (positive in 83%) and the assessment of risk factors such as residence in endemic regions (100%), particularly rural areas (reported in 36%), consumption of unpasteurized milk products (70%) and occupational exposure (35%). All patients received (combined) antibiotic treatment. However, necrotising orchitis requiring orchiectomy occurred in 5% of patients as a result of local non-response [[41], [42], [43],^45^].

Brucella epididymo-orchitis was initially mistaken for common epididymo-orchitis in 17% of cases. Genitourinary manifestation of Brucellosis is a substantial differential diagnosis for acute scrotal pain in endemic regions and therapy should be initiated without delay. There is a potential to reduce fertility. Persistent oligospermia and aspermia have been reported after Brucella epididymo-orchitis [43] but substantial data regarding this field is lacking.

### 3.7 Thrombosis of the spermatic vein or pampiniform plexus

Seven articles on thrombosis of the spermatic vein or pampiniform plexus were identified (Table 1). In five cases, thrombosis was pretherapeutically diagnosed by ultrasound as described in Table 3. Three patients received conservative treatment (analgesics, antibiotics and/or therapeutic anticoagulation and scrotal elevation). Five patients underwent excision of the thrombosed venous segment as a result of diagnostic surgical exploration and/or due to non-response to conservative treatment [[46], [47], [48], [49]].

Main factors that contribute to the formation of thrombosis are blood stasis, hypercoagulable status and mural factors. Predominantly left-sided occurrence might be associated with the same factors that contribute to varicocele formation: Perpendicular junction of the spermatic vein into the left renal vein and insufficient venal valves. Presence of varicocele, protein C deficiency, infective status, drug abuse and being sedentary were identified as predisposing factors for spermatic vein thrombosis in our analysis. Conservative therapy is a primary, testicle-sparing option, surgical excision of the thrombosed segment has been described as an option for patients unresponsive to conservative treatment.

### 3.8 Acute aortic syndrome

Five articles on acute scrotal pain in patients with acute aortic syndrome were identified (Table 1). Four of eight patients presented with scrotal pain before signs suggestive for abdominal aortic aneurysm rupture occurred [[50], [51], [52], [53]]. No patient showed local changes of the scrotum. Scrotal pain in acute aortic syndrome is likely the result of compression of the ilioinguinal/genitofemoral nerve by aortic aneurysma/retroperitoneal hematoma, also described as “referred” pain, acting at a site distant from the actual disease [52].

### 3.9 Acute appendicitis

We identified five cases of **acute appendicitis** causative for acute scrotal pain [[54], [55], [56], [57], [58]] (Table 1). For two patients scrotal pain was the first and leading symptom [54,55]. In two cases inflamed appendix was herniated through the inguinal canal into the scrotum [57,58].

### 3.10 Other rare causes of acute scrotal pain

Other rare causes are described in Table 2. **Inguinal hernia** with the presence of scrotoinguinal mass, one of them resulting in testicular ischemia, was identified as potential source of acute scrotal pain [59,60]. Patients with **tuberculous epididymo-orchitis** [61,62] and **tuberculosis of the tunica albuginea** were initially treated for unspecific epididymo-orchitis but underwent scrotal exploration due to progressive disease [63]. Microbiologic and pathologic specimen revealed tuberculosis. Patients received antituberculous treatment and recovered well. Patients with scrotal **filariasis** underwent scrotal exploration under the suspicion of testicular torsion [64,65]. Presence of filariae was detected upon pathologic examination. Patients recovered well under filaricide systemic treatment. All cases with filariasis and tuberculosis were originated from endemic countries suggesting the role of careful medical history taking. Ultrasound imaging in **emphysematous epididymo-orchitis** provoked by gas-producing bacteria showed hyperechogenic, highly reflective foci suggestive of gas collection within the scrotum (Table 3) [66,67]. Patients received orchiectomy and debridement in order to avoid progression to necrotising fasciitis. **Ureteral** **stone** has the potential to cause scrotal pain. Impacted stone and adjacent inflammation at a localization where the ureter crosses over the genitofemoral nerve is considered causative for referred scrotal pain [52,68].

### 3.11 Strength and limitations of the review and perspectives

The strength of our review is the systematic approach applied to the analysis of literature. Two authors independently performed article selection in order to minimize the risk for selection bias. Disease entities were by definition of low incidence, so predominantly case reports/series or retrospective cohort studies were found. To our knowledge, higher quality studies regarding this topic are not available. Case reports are vulnerable to bias in patient selection and reporting. So the potential to draw a treatment algorithm is limited by the weakness of the underlying studies. For the future, well-designed studies are worthwhile.

Nevertheless, valuable information has been collected that enables us to draw important conclusions for the clinician. We herein present a profile of rare causes of acute scrotal pain in order to provide knowledge and recognition of the differential diagnoses.

## 4 Conclusions

With our literature review we identified rare differential diagnoses of acute scrotal pain that have the potential to be managed conservatively once a malignant tumor or testicular torsion is excluded. We encourage to explore the full medical history and perform a comprehensive physical examination. Scrotal ultrasound is an indispensable tool for recognition of a correct diagnosis. Although organ-sparing approach should be provided whenever feasible, surgical exploration is indicated in case of doubt. If available, an intraoperative frozen section can help to determine whether an orchiectomy is indicated. In cases that are managed conservatively it seems crucial to re-evaluate the indication for surgery throughout the disease course as complications requiring intervention may occur. If concomitant symptoms indicate systemic disease or acute abdomen, the respective specialty should be involved in order to provide organ- and sometimes life-sparing procedures.

## Ethical approval

Not applicable.

## Author contribution

**Nadine Sieger:** Conceptualization; data collection; data analysis and interpretation; drafting, revision and approval of final manuscript.

**Francesca Di Quilio:** Conceptualization; data collection.

**Jens-Uwe Stolzenburg:** Revision and approval of final manuscript.

## Funding

None.

## Research registration Unique Identifying number (UIN)

1 Name of the registry: PROSPERO
2 Unique Identifying number or registration ID: CRD42018099472
3 Hyperlink to the registration (must be publicly accessible):

http://www.crd.york.ac.uk/PROSPERO/display_record.php?ID=CRD42018099472

## Guarantor

Nadine Sieger.

## Provenance and peer review

Not commissioned, externally peer reviewed.

## Declaration of competing interest

None declared.
